# Tetrel Interactions from an Interacting Quantum Atoms Perspective

**DOI:** 10.3390/molecules24122204

**Published:** 2019-06-12

**Authors:** José Luis Casals-Sainz, Aurora Costales Castro, Evelio Francisco, Ángel Martín Pendás

**Affiliations:** Departamento de Química Física y Analítica, Julián Clavería, 6, Facultad de Química, Universidad de Oviedo, 33006 Oviedo, Spain; jluiscasalssainz@gmail.com (J.L.C.-S.); costalesmaria@uniovi.es (A.C.C.); evelio@uniovi.es (E.F.)

**Keywords:** energy partition, interacting quantum atoms, quantum theory of atoms in molecules, delocalization index, covalent interaction, self-energy

## Abstract

Tetrel bonds, the purportedly non-covalent interaction between a molecule that contains an atom of group 14 and an anion or (more generally) an atom or molecule with lone electron pairs, are under intense scrutiny. In this work, we perform an interacting quantum atoms (IQA) analysis of several simple complexes formed between an electrophilic fragment (A) (CH_3_F, CH_4_, CO_2_, CS_2_, SiO_2_, SiH_3_F, SiH_4_, GeH_3_F, GeO_2_, and GeH_4_) and an electron-pair-rich system (B) (NCH, NCO^-^, OCN^-^, F^-^, Br^-^, CN^-^, CO, CS, Kr, NC^-^, NH_3_, OC, OH_2_, SH^-^, and N_3_−) at the aug-cc-pvtz coupled cluster singles and doubles (CCSD) level of calculation. The binding energy ($E_{\mathrm{bind}}^{\mathrm{AB}}$) is separated into intrafragment and inter-fragment components, and the latter in turn split into classical and covalent contributions. It is shown that the three terms are important in determining $E_{\mathrm{bind}}^{\mathrm{AB}}$, with absolute values that increase in passing from electrophilic fragments containing C, Ge, and Si. The degree of covalency between A and B is measured through the real space bond order known as the delocalization index ($\delta^{\mathrm{AB}}$). Finally, a good linear correlation is found between $\delta^{\mathrm{AB}}$ and $E_{\mathrm{xc}}^{\mathrm{AB}}$, the exchange correlation (xc) or covalent contribution to $E_{\mathrm{bind}}^{\mathrm{AB}}$.

## 1. Introduction

There has been a growing interest in the last years in the field of non-covalent interactions (NCI) [1], which have been shown to be critical for the correct description of the structure and properties of different molecules and materials [2,3], including nanomaterials [4,5], molecular solids [6,7], surfaces [8,9], and biological systems [10,11,12]. From the many types of interactions that are usually classified as non-covalent, hydrogen bonding A−H···D [13,14], where A is a group more electronegative that H and D is an entity able to act as an electron donor, is undoubtedly the best-known by all chemists. Besides hydrogen and halogen bonding [15,16] (possibly the best-known type of NCI after the former), other purported NCIs involving atoms of groups 14, 15, and 16 (and even rare gas atoms [17,18]) have recently received the names of tetrel, pnictogen [19,20], and chalcogen bonding, respectively, although some of these complexes were identified and characterized by different experimental techniques long before they were given these names [19]. In all of them, the 14, 15, or 16 group element, acting as an electron acceptor or electrophilic site, seeks the nucleophilic part of another system, for instance an atomic or molecular anion (F^-^,Br^-^,⋯,CN^-^, NC^-^,N_3_−,⋯), a π− electron pair of a Lewis base, or a non-bonding electron pair of an arbitrary molecule. As far as tetrel bonds are concerned, and to name just a few works, Bürgi et al. pioneered the study and description of nucleophilic additions to carbonyl C-atoms or $n\to \pi^{⋆}$ interactions [21,22,23], recognized by several authors as important to biology [24,25,26], and Thomas et al. found experimental evidence for carbon bonding (an interaction where a carbon atom acts as an electrophilic site toward a variety of nucleophiles) in the solid state from X-ray charge density analysis [27]. Southern and Bryce presented results for NMR parameters of a series of model compounds in which a tetrel bond between a methyl C atom and the N or O atom of several functional groups is found [28]. Scilabra has shown that contacts between a Ge or Sn atom with different lone-pair-possessing atoms in crystal structures are quite common [29], and Mitzel and Losehand found crystal structures of Si(ONMe${}_{2}$)${}_{4}$ with a short-distance Si–N bond [30]. A particularly relevant mention is also the work of Bauzá, Frontera, and Mooibroek, who pioneered tetrel interactions and wrote several interesting reviews on the topic [31,32,33,34,35].

From the theoretical side, Scheiner [36] made a comparison of halide receptors based on H, halogen, chalcogen, pnicogen, and tetrel bonds by means of molecular electrostatic potential (MEP) maps and natural bond orbital (NBO) analyses [37,38] at the density functional theory (DFT) M06-2X//aug-cc-pDVZ level of calculation, and Alkorta et al. performed MP2//aug-cc-pVTZ energetic studies, calculating harmonic vibrational frequencies, EOM-CCSD spin-spin coupling constants, and NBO analyses in complexes of CO_2_ with azoles [39], azines [40], and carbenes as electron pair donors to CO_2_ [40]. Carbon bonding in the X−C···Y (X=O/F, Y=O/S/F/Cl/Br/N/P) and X−C··· (X=F,Cl,Br,CN) systems were studied by Mani and Arunan [41,42], started with MP2/6-311+G(3df,2p) and MP2//aug-cc-pvtz calculations to optimize the geometry, and followed this up with CCSD(T) calculations to estimate the interaction energy and NBO, quantum theory of atoms in molecules (QTAIM) [43,44], and MEP analyses. Interestingly, the formation of the type of tetrel interaction called carbon bonding had been previously proposed by Grabowski as a preliminary step necessary in SN${}_{2}$ reactions [45].

Mixed theoretical/experimental studies have also been carried out by Sethio, Oliveira, and Kraka, aimed at a quantitative assessment of tetrel bonding utilizing vibrational spectroscopy [46]. Finally, several other theoretical papers have been published in recent years to determine the influence that a substitution of the ligands have on the tetrel bond strength [47,48,49,50,51,52,53].

As far as we know, in the already extensive literature existing today regarding theoretical studies of tetrel bonding systems, there is no publication in which a detailed energy partition analysis of these compounds has been performed. We will carry out this study in this work. Specifically, we will use the interacting quantum atoms (IQA) method [54,55,56,57] to analyze about thirty complexes formed between an electrophilic fragment of the set CH_3_F, CH_4_, CO_2_, CS_2_, SiO_2_, SiH_3_F, SiH_4_, GeH_3_F, GeO_2_, and GeH_4_ and an electron-pair-rich system of the set NCH, NCO^-^, OCN^-^, F^-^, Br^-^, CN^-^, CO, CS, Kr, NC^-^, NH_3_, OC, OH_2_, SH^-^, and N_3_−. IQA is a real space orbital invariant energy partition method inspired by the QTAIM that exactly recovers the total energy of a molecule by splitting its total energy in terms of intra-atomic and interatomic components. It is general in the sense that any type of wavefunction may in principle be analyzed with it. All that is required is to have at our disposal the one- and (diagonal) two-particle density matrices. Hartree–Fock, complete active space (CAS), full-CI, CCSD and EOM-CCSD wavefunctions have been analyzed to date using IQA. DFT calculations can also be used within the IQA partition, at least formally, taking the Kohn–Sham determinant of the system as the approximate wavefunction and performing a physically sound scaling of the interactions [58,59].

The degree of detail with which IQA allows us to scrutinize the energetic interactions is really high. However, in this work we will not use all of these potentialities. This means that we will only split the different energy contributions at the fragment, and not the atomic level. We will thus worry neither about analyzing how the net energy of a given fragment is distributed among its atoms nor on how the atoms of this fragment interact with each other. Discussions related to the geometry of the fragments will not be considered either. Once the geometry of the molecules has been optimized (as discussed in the next section), all subsequent energetic analyses will refer to these geometries.

The rest of the article has been divided as follows. The theoretical methods used in our analyses are briefly discussed in Section 2. Some computational details related to the above methods are given in Section 3. The results and their discussion are presented in Section 4. Finally, the more relevant conclusions of this work are given in Section 5.

## 2. Theoretical Methods

In this section, we describe very briefly the interacting quantum atoms (IQA) [54,55,56,57] approach that has been used to obtain all the data and energetic quantities that will be discussed in Section 4. We also give some relevant details of the coupled cluster method up to single and double excitations (CCSD) [60] that we have employed to derive the wavefunctions that are fed into the IQA method, and comment, also very briefly, on some points regarding the computation, within the IQA scheme, of the binding energy of a supermolecule AB from its IQA energetic quantities and those of the isolated fragments *A* and *B*.

The interacting quantum atoms (IQA) method is an energy partition scheme that is based on the exhaustive partition of the real space occupied by a molecule according to the quantum theory of atoms in molecules (QTAIM) [43]. IQA exactly recovers the total energy of a molecule and can be applied, in principle, to any level of theory as soon as the one-particle, $\rho_{1}\left(r_{1},r_{1}^{\prime }\right)$, and (diagonal) two-particle, $\rho_{2}\left(r_{1},r_{2}\right)$, density matrices are available. The total electronic Born–Oppenheimer energy of a molecule reads as [61]

(1) $$E=\int \hat{h}\rho_{1}\left(r_{1},r_{1}^{\prime }\right)dr_{1}+\frac{1}{2}\;\int \int \;\frac{\rho_{2}\left(r_{1},r_{2}\right)}{r_{12}}dr_{1}dr_{2}+E_{\mathrm{nuc}},$$

where $\hat{h}$ is the monoelectronic operator that includes the kinetic energy and nuclear attraction terms, $\hat{h_{i}}={\hat{t}}_{i}-\sum_{A}Z_{A}/r_{iA}$, and $E_{\mathrm{nuc}}=\sum_{A>B}Z_{A}Z_{B}R_{AB}^{-1}$ is the total nuclear repulsion energy. If the physical space $R^{3}$ is partitioned according to QTAIM [43,44], $R^{3}=\cup_{A}\Omega_{A}$, where $\Omega_{A}$ represents the atomic basin of atom *A*, it is clear that the monoelectronic energy in Equation (1) can be split into as many contributions as the total number of atoms of the molecule (say *n*), and the bielectronic energy into $n^{2}$ terms. Doing so, we obtain the IQA energy partition

$$E=\sum_{A}\int_{\Omega_{A}}\hat{h}\rho_{1}\left(r_{1},r_{1}^{\prime }\right)dr_{1}+\frac{1}{2}\sum_{A,B}\int_{\Omega_{A}}\int_{\Omega_{B}}\frac{\rho_{2}\left(r_{1},r_{2}\right)}{r_{12}}dr_{1}dr_{2}+E_{\mathrm{nuc}}.$$

Grouping together intra- (A=B) and inter-atomic (A≠B) terms,

(2) $$\begin{array}{ccc} E & = & \sum_{A}E_{\mathrm{net}}^{A}+\sum_{A>B}E_{\mathrm{int}}^{\mathrm{AB}} \end{array}$$

(3) $$\begin{array}{cc} = & \sum_{A}T^{A}+V_{\mathrm{ne}}^{\mathrm{AA}}+V_{\mathrm{ee}}^{\mathrm{AA}}+\sum_{A>B}V_{\mathrm{nn}}^{\mathrm{AB}}+V_{\mathrm{ne}}^{\mathrm{AB}}+V_{\mathrm{ne}}^{\mathrm{BA}}+V_{\mathrm{ee}}^{\mathrm{AB}}, \end{array}$$

where $E_{\mathrm{net}}^{A}$ is the net or self-energy of atom *A*, which collects all the energy terms involving exclusively the nucleus and electrons within this atom, and $E_{\mathrm{int}}^{\mathrm{AB}}$ is the total interatomic energy between atoms *A* and *B*. In Equation (3), $V_{\mathrm{nn}}^{\mathrm{AB}}$, $V_{\mathrm{ne}}^{\mathrm{AB}}$, and $V_{\mathrm{ee}}^{\mathrm{AB}}$ when A≠B represent the nucleus–nucleus, nucleus–electron, and electron–electron interactions associated to the pair of atoms A,B, and $V_{\mathrm{ne}}^{\mathrm{AA}}$ is the interaction of the electrons inside $\Omega_{A}$ with the nucleus of this atomic basin. $V_{\mathrm{ee}}^{\mathrm{AA}}$ is the total electron repulsion of the electrons inside $\Omega_{A}$ among themselves. When *A* or *B* or both represent groups of atoms or molecules instead of single atoms, Equations (2) and (3) remain almost valid, with minor modifications that involve only the self-energy term $E_{\mathrm{net}}^{A}$ [62]. By splitting $\rho_{2}\left(r_{1},r_{2}\right)$ into its classical and exchange-correlation components, $E_{\mathrm{int}}^{\mathrm{AB}}$ may be written as $E_{\mathrm{int}}^{\mathrm{AB}}=V_{\mathrm{cl}}^{\mathrm{AB}}+V_{\mathrm{xc}}^{\mathrm{AB}}$, where $V_{\mathrm{cl}}^{\mathrm{AB}}$ is the electrostatic interaction between all particles (nuclei plus electrons) inside *A* with all particles inside *B*, and $V_{\mathrm{xc}}^{\mathrm{AB}}$ represents the purely quantum-mechanical or covalent interaction. In the Hartree–Fock (HF) approximation, only the Fermi correlation is taken into account, which leads to $V_{\mathrm{xc}}^{\mathrm{AB}}=V_{x}^{\mathrm{AB}}$ in the IQA method, which contains only exchange interactions. However, for many correlated methods it is still possible to write formally $V_{\mathrm{xc}}^{\mathrm{AB}}=V_{x}^{\mathrm{AB}}+V_{\mathrm{corr}}^{\mathrm{AB}}$, provided that the pure-exchange two-particle density matrix, $\rho_{2}^{x}\left(r_{1},r_{2}\right)$, is taken as the Dirac–Fock expression $\rho_{2}^{x}\left(r_{1},r_{2}\right)=-\rho_{1}\left(r_{1},r_{2}\right)\times \rho_{1}\left(r_{2},r_{1}\right)$, and $\rho_{2}^{\mathrm{corr}}\left(r_{1},r_{2}\right)$ is simply defined as $\rho_{2}^{\mathrm{corr}}\left(r_{1},r_{2}\right)=\rho_{2}^{\mathrm{xc}}\left(r_{1},r_{2}\right)-\rho_{2}^{x}\left(r_{1},r_{2}\right)$. Among the several post-HF levels of theory including dynamical correlation energy contributions (absolutely necessary to address the study of the systems considered in this work), we have chosen the coupled cluster method including only single and double excitations [60]. Other approaches, such as the second-order Møller–Plesset perturbation theory (MP2) [63], which overestimates the dispersion energy interactions [64], have not been considered. In the CCSD method, the reference wavefunction of the system is the HF determinant, and the total energy *E* is written as the sum of the energy of this reference wavefunction plus the correlation energy of the molecule, $E=E_{\mathrm{HF}}+E_{\mathrm{corr}}$. The latter can be expressed in terms of the CCSD amplitudes $t_{ij}^{ab}$ as

(4) $$E_{\mathrm{corr}}=\sum_{iajb}t_{ij}^{ab}\left(ia|jb\right),$$

where (ia|jb) are two electron integrals in the molecular orbital basis (MO), the Mulliken convention has been used, and i,j and a,b refer to occupied and virtual orbitals, respectively. As the total energy *E* itself, $E_{\mathrm{corr}}$ can be partitioned à la IQA, leading to intra-atomic, $E_{\mathrm{corr}}^{\mathrm{net},A}$, and interatomic, $E_{\mathrm{corr}}^{\mathrm{int},\mathrm{AB}}$ terms. Other details of the IQA implementation within the CCSD method are described elsewhere [65].

The binding energy between two atoms, fragments or molecules *A* and *B* is defined by

(5) $$E_{\mathrm{bind}}^{\mathrm{AB}}=E^{\mathrm{AB}}-E^{A}-E^{B},$$

where $E^{\mathrm{AB}}$, $E^{A}$, and $E^{B}$ are the total energies of AB, *A*, and *B*, respectively. If these three total energies are separated into their HF and correlation components, $E_{\mathrm{bind}}^{\mathrm{AB}}$ results:

(6) $$\begin{array}{ccc} E_{\mathrm{bind}}^{\mathrm{AB}} & = & \left(E_{\mathrm{HF}}^{\mathrm{AB}}-E_{\mathrm{HF}}^{A}-E_{\mathrm{HF}}^{B}\right)+\left(E_{\mathrm{corr}}^{\mathrm{AB}}-E_{\mathrm{corr}}^{A}-E_{\mathrm{corr}}^{B}\right) \end{array}$$

(7) $$\begin{array}{ccc} & = & E_{\mathrm{HF},\mathrm{bind}}^{\mathrm{AB}}+E_{\mathrm{corr},\mathrm{bind}}^{\mathrm{AB}}. \end{array}$$

Assuming that the geometries of *A* and *B* are the same as in the supermolecule AB, $E_{\mathrm{bind}}^{\mathrm{AB}}$ in the IQA method is given by

(8) $$\begin{array}{ccc} E_{\mathrm{bind}}^{\mathrm{AB}} & = & E_{\mathrm{def}}^{A}+E_{\mathrm{def}}^{B}+V_{\mathrm{cl}}^{\mathrm{AB}}+\left(V_{x}^{\mathrm{AB}}+E_{\mathrm{corr}}^{\mathrm{AB}}\right) \end{array}$$

(9) $$\begin{array}{ccc} & = & E_{\mathrm{def}}^{A}+E_{\mathrm{def}}^{B}+V_{\mathrm{cl}}^{\mathrm{AB}}+V_{\mathrm{xc}}^{\mathrm{AB}}, \end{array}$$

where each deformation energy $E_{\mathrm{def}}^{R}=E_{\mathrm{net}}^{R}-E^{R}$ (R=A,B) represents the energy change suffered by R when it passes from being isolated to interacting with the other fragment(s). In case the geometry of *R* has changed in going from the isolated state to the supermolecule, the so-called preparation energy, $E_{\mathrm{prep}}^{R}$, defined as $E_{\mathrm{prep}}^{R}=E^{R}\left(\mathrm{supermolecule}\;\mathrm{geometry}\right)-E^{R}\left(\mathrm{isolated}\;\mathrm{geometry}\right)$, must be added to $E_{\mathrm{bind}}$. On the other hand, it is customary in some energy partition methods, such as the energy decomposition analysis (EDA) method [66,67,68], to associate the term of Pauli exchange-repulsion (xr) with the increase of energy that takes place as a consequence of the antisymmetrization and normalization of the direct product of fragments’ wavefunctions. Here, however, we will reserve this name to the sum of $E_{\mathrm{def}}^{A}$, $E_{\mathrm{def}}^{B}$, and $V_{x}^{\mathrm{AB}}$, i.e.,

(10) $$E_{\mathrm{xr}}^{\mathrm{AB}}=E_{\mathrm{def}}^{A}+E_{\mathrm{def}}^{B}+V_{x}^{\mathrm{AB}}.$$

Clearly, the origin of $E_{\mathrm{xr}}^{\mathrm{AB}}$ in IQA is strictly different from that in EDA. However, in spite of this, the IQA $E_{\mathrm{xr}}^{\mathrm{AB}}$ energy corresponds, in many ways, to other conventional exchange-repulsion terms [57,69]. For instance, as in other schemes, this energy turns out to be usually (but not necessarily) positive (see below). For that reason, we have decided to keep the name of exchange-repulsion for the energetic term defined in Equation (10). After using this definition in Equation (8), $E_{\mathrm{bind}}$ takes the form

(11) $$E_{\mathrm{bind}}^{\mathrm{AB}}=V_{\mathrm{cl}}^{\mathrm{AB}}+E_{\mathrm{xr}}^{\mathrm{AB}}+E_{\mathrm{corr}}^{\mathrm{AB}}.$$

In all the calculations presented in the following section, the basis set superposition error (BSSE), inherent to the calculation $E_{\mathrm{bind}}$, has been corrected in the IQA scheme by using the Boys and Bernardi counterpoise method [70] to compute the total energies of the isolated fragments, $E^{R}$.

## 3. Computational Details

The calculations of this work have been done as follows. In a first step, the geometries of all the studied systems were optimized at the density functional theory (DFT) level with the WB97X-D functional [71] and the aug-cc-pvtz basis set [72] using the gamess package [73]. Then, single-point CCSD calculations at the optimized geometries were carried out with a locally modified copy of the PySCF code [74] using the same basis set. Core orbitals were frozen, and for truncating the virtual space, the frozen natural orbital approximation (FNO) with a cutoff in the natural occupations of $10^{-4}$ was used [75]. All the interaction energies include the BSSE correction [70]. The CCSD amplitudes $t_{ij}^{ab}$ and the one- and two-particle density matrices were also obtained with PySCF [74].

The IQA energy partitioning was performed with our in-house program promolden [76]. The necessarily numerical IQA integrations were done using β-spheres for all the atoms, with radii between 0.1 and 0.3 bohr. Restricted angular Lebedev quadratures with 3074 points and 451-point Gauss–Chebyshev mapped radial grids were used inside the β-spheres, with *L* expansions cut at l=8. Outside the β-spheres, extended 5810-point Lebedev, 551 mapped radial point Gauss–Legendre quadratures, and *L* expansions up to l=10 were selected.

## 4. Results and Discussion

**Figure 1:**
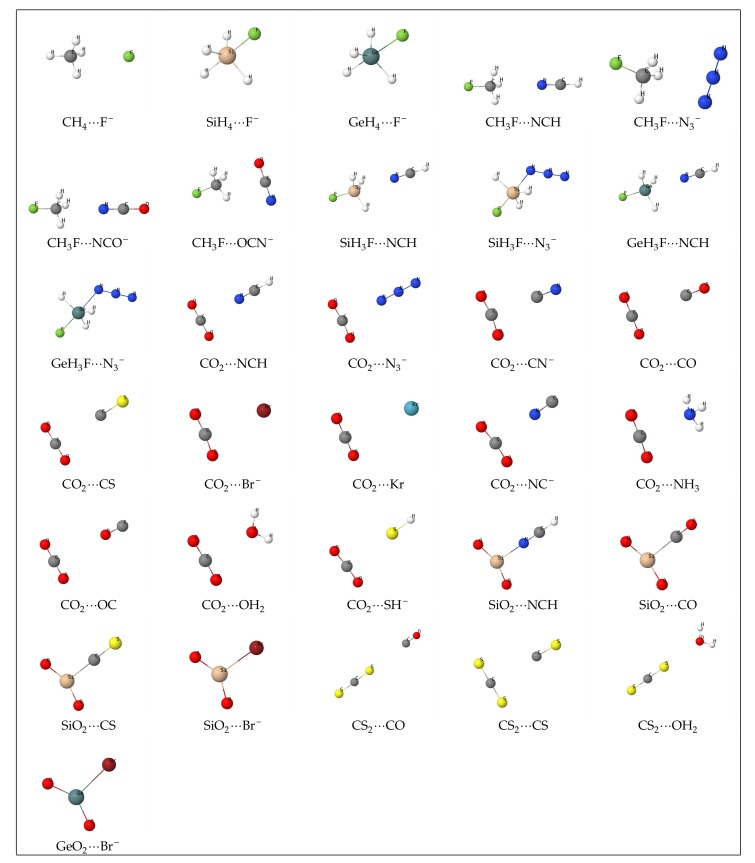
AB complexes studied in this work.

A graphical rendering of the optimized complexes studied in this work appears in Figure 1. The full set of atomic Cartesian coordinates is collected in the supplementary information. Since we have not carried out a systematic exploration of all possible local energy minima, there is no guarantee that the geometry depicted in the figure corresponds to the global minimum. For nine of the 31 complexes, both fragments are connected by a solid line (SiH_4_···F^-^, GeH_4_···F^-^, SiH_3_F···N_3_−, GeH_3_F···N_3_−, SiO_2_···NCH, SiO_2_···CO, SiO_2_···CS, SiO_2_···Br^-^, GeO_2_···Br^-^). Under the rendering conditions that we have used, this implies that the two linked atoms are separated by a distance less than the sum of their covalent radii plus 0.025 Å. In the remaining 22 systems, there is no connection line between any pair of atoms *a* ∈ A and *b* ∈ B. As we will see, the nine connected complexes are those with a delocalization index $\delta^{\mathrm{AB}}$ (a measure of the covalent bond order in real space, see Table 2) greater than 0.5, similar to that of a typical polar covalent bond. It seems that, other factors aside, a clear correlation exists between $\delta^{\mathrm{AB}}$ and the distance between the connected atoms of both fragments.

Given the numerical character of all the IQA integrated quantities [77,78], we want to check, first of all, the reliability and consistency of our results. We collect in Table 1 the binding energy of the different A···B systems computed directly as the total CCSD energy of the dimer, $E^{\mathrm{AB}}$, minus the sum of the CCSD energies of both monomers, $E^{A}$ and $E^{B}$ ($E_{\mathrm{bind}}$(CCSD), Equation (5)), the IQA binding energy obtained from Equation (8) ($E_{\mathrm{bind}}$(IQA)), its difference (diff), and the total IQA integrated charges, $Q_{A}$, $Q_{B}$, and $Q=Q_{A}+Q_{B}$. Almost systematically, the exact value of *Q* is very well reproduced by our IQA integrations. In all of the systems except GeH_3_F···N_3_−, CH_3_F···N_3_− and SiO_2_···Br^-^, the error is less than or equal to 0.001 e. As binding energies are regarded, the absolute error is lower than 0.5 Kcal/mol in 20 of the 30 systems and greater than this number in the remaining cases. Although it cannot be inferred from the numbers in the table, it can be said that almost 100% the error associated with the computation of $E_{\mathrm{bind}}$(IQA) is due to its HF contribution, $E_{\mathrm{HF},\mathrm{bind}}$(IQA) (see Equation (7)), since the IQA integrations of $E_{\mathrm{corr}}^{\mathrm{AB}}$, $E_{\mathrm{corr}}^{A}$, and $E_{\mathrm{corr}}^{B}$ (see Equation (6)) reproduce their CCSD-analogous quantities extraordinarily well. Be that as it may, the fact that the CO_2_···Kr system in the IQA partition, contrarily to the CCSD calculation, is predicted to be unstable with respect to the isolated fragments, should not be taken too seriously given that the difference between $E_{\mathrm{bind}}$(IQA)) and $E_{\mathrm{bind}}$(CCSD)) in this system (0.52 Kcal/mol) is comparable to the average error of the numerical IQA integrations. Nonetheless, we believe that the present results are overall quite satisfactory, although we do not want to deny that the weakest point of the IQA energy partitioning method lies possibly in the existing difficulties of further reducing the errors associated with the numerical integrations of the method.

The electronic and total (electronic plus nuclear) charge of every atom (or fragment) of a molecular system, obtained by integrating the electronic or total density inside the corresponding atomic (or fragment) basin, is one of the main outcomes of the QTAIM methodology. It can be seen in Table 1 that the electronic charge transferred from fragment B to fragment A is relatively small in all of the studied systems for which the isolated fragment B is neutral. Only in CO_2_···NH_3_, SiO_2_···CO, and SiO_2_···NCH is this transfer greater than 0.01 e, whereas in the cases of CO_2_···OC and CS_2_···CS, both *A* and *B* remain almost neutral after the complex is formed from the isolated fragments. Conversely, when the isolated fragment B is negatively charged, its ability to transfer electrons to the acceptor fragment increases notably. The most representative examples of this behavior are the systems GeH_3_F···N_3_−, GeO_2_···Br^-^, GeH_4_···F^-^, and SiO_2_···Br^-^. Given the generally greater polarizability of the valence electrons in anions as compared to neutral molecules, this is not a surprising result. Regarding the azide anion N_3_−, we observe in Table 1 how its ability to transfer electrons to the acceptor fragment MH_3_F (M=C,Si,Ge) increases on descending in a group. The gain of electrons by the SiO_2_ fragment in the three of the four systems of Table 1 in which this fragment appears is also greater than that corresponding to the CO_2_ molecule in the equivalent complexes. Only in CO_2_···CS is the CO_2_ molecule more negatively charged than the SiO_2_ molecule in SiO_2_···CS.

We will analyze now the different energetic contributions to the binding energy of the studied complexes. A first point to remark is that the electron relaxation that takes place within the A and B fragments when they pass from the isolated state to their final position in the supermolecule leads systematically to positive values of the deformation energies. This behavior is general whenever the net energies of A and B in the isolated state ($E^{A}$, $E^{B}$) and in the supermolecule ($E_{\mathrm{net}}^{A}$, $E_{\mathrm{net}}^{B}$) are computed with the same electronic structure method and there is no charge transfer from A to B or from B to A. In the present calculations, we have seen that this transfer is actually very small (except in the very few cases cited above where the isolated fragment B is an anion and, even in these cases, we have seen that the B→A electron transfer is not too large). Hence, the electronic reorganization that takes place when the supermolecule is formed from the isolated fragments is always accompanied by an increase in the deformation energy contribution to the binding energy.

There is no general rule to uncover which of the two deformation energies, $E_{\mathrm{def}}^{A}$ or $E_{\mathrm{deb}}^{B}$, is the dominant of the two in each system. Actually, there seems to be a tendency for both fragments to have deformation energies of a similar magnitude. For a given acceptor fragment A, its deformation energy obviously depends on the companion donor fragment B. With the exception of CO_2_···SH^-^, $E_{\mathrm{def}}^{CO_{2}}$ in the CH_3_F···B and CO_2_···B supermolecules is much greater when the isolated fragment B is negative than when it is neutral. This result is not at all surprising, for it seems reasonable to think that the ability of B to alter the electronic distribution of A is greater in the first case than in the second.

Another point that is worth noting is that the ability of a given donor fragment B to alter the electron distribution of A (and consequently, to increase its deformation energy) increases in the order C > Ge > Si, where M=(C, Si, Ge) is the atom of group 14 included in fragment A. This can be easily seen in Table 1 by analyzing the deformation energy of A in the series CH_3_F···N_3_−→SiH_3_F···N_3_−→GeH_3_F···N_3_−, CH_4_···F^-^ →SiH_4_···F^-^→ GeH_4_···F^-^, and CO_2_···Br^-^ →SiO_2_···Br^-^→ GeO_2_···Br^-^. An exception of this rule is the series CH_3_F···NCH →SiH_3_F···NCH→ GeH_3_F···NCH, in which the deformation energy of the SiH_3_F fragment is marginally smaller than that of the GeH_3_F fragment. The deformation energy of the donor fragment B follows the same order.

The classical interaction between the fragments A and B, $V_{\mathrm{cl}}^{\mathrm{AB}}$, is always stabilizing. The range of values of this energetic interaction goes from almost negligible in some systems (e.g., −0.1 Kcal/mol in CO_2_···Kr and CS_2_···CO) up to a few tens of Kcal/mol in other cases. As expected, given that the point charge interaction is generally the most important contribution to $V_{\mathrm{cl}}^{\mathrm{AB}}$ and that it usually dominates over all the higher-order multipolar interactions, this classical interaction tends to be more negative when both fragments are significantly charged. There are, however, several cases in which this statement is not fulfilled at all. For instance, $V_{\mathrm{cl}}^{\mathrm{AB}}$ in the SiO_2_···CS system takes a value as large as −108 Kcal/mol, despite the fact that the absolute value of the charges of the SiO_2_ and CS fragments are smaller than 0.01 e. In other complexes, such as CO_2_···CN^-^ and CO_2_···Br^-^, the situation is the opposite. In these two cases, both fragments have a non-negligible charge, and the classical interaction between them is, however, relatively small. These facts suggest that in many of the studied systems, $V_{\mathrm{cl}}^{\mathrm{AB}}$ has important multipolar contributions and that nothing conclusive can be said about the magnitude of this energetic component looking exclusively at the values of the net charges of both fragments. The second intergroup contribution to $E_{\mathrm{bind}}^{\mathrm{AB}}$ and $E_{\mathrm{int}}^{\mathrm{AB}}$ is the exchange-correlation interaction energy, $V_{\mathrm{xc}}^{\mathrm{AB}}$. Its exchange contribution, $V_{x}^{\mathrm{AB}}$, also appears in Table 1, and the difference between both quantities gives the intergroup correlation binding energy, $E_{\mathrm{corr}}^{\mathrm{AB}}$. The comparison between $V_{\mathrm{xc}}^{\mathrm{AB}}$ and $V_{x}^{\mathrm{AB}}$ indicates that $E_{\mathrm{corr}}^{\mathrm{AB}}$ is, in general, rather small and, of course, much less important than either of them. This does not mean that the intergroup correlation energy in some of the systems is not comparable to the value of the binding energy itself: $E_{\mathrm{bind}}^{\mathrm{AB}}$ comes from the sum of several quantities, some of them possibly quite large, but the final result can be very small and comparable to one or more of the quantities that have been added.

Regarding the values of $V_{\mathrm{xc}}^{\mathrm{AB}}$ (or $V_{x}^{\mathrm{AB}}$), we must note that, similarly to $V_{\mathrm{cl}}^{\mathrm{AB}}$, the exchange-correlation energy is always a stabilizing contribution to the binding energy of the complex. In fact, the absolute values of $V_{\mathrm{xc}}^{\mathrm{AB}}$ are greater than their corresponding classical interactions in 25 of the 31 studied complexes. Five of the 6 exceptions are easy to understand as they correspond to complexes in which both fragments have relatively high charges. Only SiO_2_···CS challenges this explanation. In any case, both $V_{\mathrm{xc}}^{\mathrm{AB}}$ and $V_{\mathrm{cl}}^{\mathrm{AB}}$ are in general important in determining the final value of $V_{\mathrm{int}}^{\mathrm{AB}}$. Since the exchange-correlation interaction energy, $V_{\mathrm{xc}}^{\mathrm{AB}}$, is associated with covalency while $V_{\mathrm{cl}}^{\mathrm{AB}}$ describes ionicity, both types of interactions (covalent- and ionic-like energies) are necessary for a proper and accurate description of the complexes analyzed in the present work.

The comparison between the classical and exchange-correlation energies of Table 1 for equivalent complexes in which the central atom of the electrophilic fragment is M=C, Si, or Ge is very illuminating. For instance, for the nine AB complexes formed with A=(CH_4_, SiH_4_, GeH_4_) and B=(F^-^, N_3_−, NCH), both $V_{\mathrm{cl}}^{\mathrm{AB}}$ and $V_{\mathrm{xc}}^{\mathrm{AB}}$ increase in the order Si > Ge > C when B=F^-^ or N_3_−, while both quantities are much smaller and rather similar for the C, Si, and Ge cases when B=NCH. The explanation for this behavior is relatively simple: The M−X distance, $R_{M-X}$, where X is the atom of the donor fragment that is closer to M, decreases noticeably in the order C > Ge > Si when B=F^-^ (3.04, 2.00, and 1.76 Å, respectively) or B=N_3_− (3.03, 2.23, 2.04 Å), while $R_{M-X}$ is larger and not so different in the three cases when B=NCH (3.22, 2.98, and 2.96 Å for M=C, Ge, and Si, respectively). Thus, the value of $R_{M-X}$ determines, to a large extent, the magnitude of the classical and exchange-correlation interaction energies. (The distances between all the inequivalent atomic pairs are collected in the supplementary information.) Actually, the relative magnitudes of the deformation energies $E_{\mathrm{def}}^{A}$ and $E_{\mathrm{def}}^{B}$ for these nine complexes can also be explained based almost exclusively on the value of $R_{M-X}$. In turn, $R_{M-X}$ correlates quite well with the total charge of M, +0.14 (C), +3.12 (Si), and +2.10 (Ge) when B=F^-^ and +0.69 (C), +3.17 (Si), and +2.22 (Ge) when B=N_3_−.

**Figure 2:**
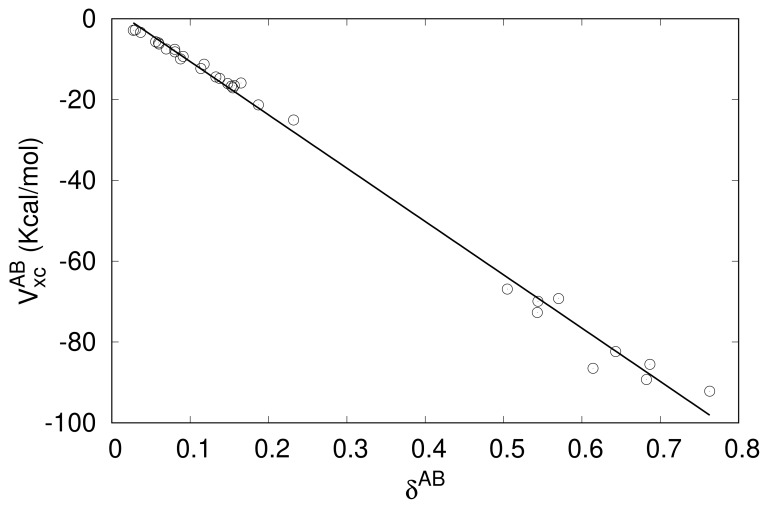
$V_{\mathrm{xc}}^{\mathrm{AB}}$ versus $\delta^{\mathrm{AB}}$ values for the complexes of Table 1.

It has been recently shown that there is a theoretical link between the conventional concept of bond order and the energetics of chemical interactions [79,80]. Expanding $V_{\mathrm{xc}}^{\mathrm{AB}}$ as a multipolar series, the zero-th order term in the expansion (that dominates $V_{\mathrm{xc}}^{\mathrm{AB}}$) is nothing but a distance-scaled bond order,

(12) $$V_{\mathrm{xc}}^{\mathrm{AB}}≃-\frac{\delta^{\mathrm{AB}}}{2R_{\mathrm{AB}}},$$

where $\delta^{\mathrm{AB}}=-2\int_{\Omega_{A}}\int_{\Omega_{B}}\rho_{2}^{\mathrm{xc}}\left(r_{1},r_{2}\right)dr_{1}dr_{2}$ is the delocalization index between the atoms A and B [81], a measure in real space of the bond order between both atoms. To explore to what extent the above equation is satisfied when A and B are fragments instead of single atoms, we have computed the $\delta^{\mathrm{AB}}$ values for the studied complexes (Table 2) and plotted $V_{\mathrm{xc}}^{\mathrm{AB}}$ versus $\delta^{\mathrm{AB}}$ in Figure 2.

Although Equation (12) is approximate even when A and B are atoms and the average distance between fragments (say $R_{\mathrm{AB}}$) can be different in each of the studied complexes, a linear correlation exists between $V_{\mathrm{xc}}^{\mathrm{AB}}$, the exchange-correlation interfragment energy, and $\delta^{\mathrm{AB}}$, the covalent bond order between these fragments [80]. The more significant deviations from the trend in the lower part of Figure 2 (high $\delta^{\mathrm{AB}}$ values) is due to two reasons. The first one is that Equation (12) is only approximate. As the fragments are formed with heavier and/or more polarizable atoms, higher multipolar contributions to $V_{\mathrm{xc}}^{\mathrm{AB}}$ become more important, making Equation (12) increasingly inaccurate. Secondly, in these cases, the multipolar expansion itself, regardless of the maximum order to which it is carried out, is no longer valid for short-range energy components (not only in $V_{\mathrm{xc}}^{\mathrm{AB}}$ but also in $V_{\mathrm{cl}}^{\mathrm{AB}}$) are essential due penetration energy contributions. Of course, the analysis of the degree of compliance of Equation (12) in the present context can be refined. For instance, labeling $a_{1},a_{2},\cdots$ and $b_{1},b_{2},\cdots$ the atoms of A and B, respectively, $V_{\mathrm{xc}}^{\mathrm{AB}}$ and $\delta^{\mathrm{AB}}$ are exactly given by $V_{\mathrm{xc}}^{\mathrm{AB}}=\sum_{i\in A}\sum_{j\in B}V_{\mathrm{xc}}^{a_{i}b_{j}}$ and $\delta^{\mathrm{AB}}=\sum_{i\in A}\sum_{j\in B}\delta^{a_{i}b_{j}}$, and the exchange-correlation energy between atoms $a_{i}$ and $b_{j}$ can be approximated as $-\delta^{a_{i}b_{j}}/\left(2R_{a_{i}b_{j}}\right)$. Hence,

(13) $$V_{\mathrm{xc}}^{\mathrm{AB}}≃-\sum_{i\in A}\sum_{j\in B}\frac{\delta^{a_{i}b_{j}}}{2R_{a_{i}b_{j}}}.$$

This equation is a computationally cheap form to approximately evaluate $V_{\mathrm{xc}}^{\mathrm{AB}}$, since the calculation of each $\delta^{a_{i}b_{j}}$ requires only a three-dimensional integration, whereas $V_{\mathrm{xc}}^{\mathrm{AB}}$ needs a six-dimensional numerical quadrature, much more complicated in all aspects.

The CCSD delocalization indexes in Table 2 range from very small values, highlighting that the interaction between fragments A and B is basically non-covalent, up to values well above 0.5, which are typical of some prototype polar-covalent single bonds. These latter values occur specifically in complexes in which the electron acceptor fragment contains the Si or Ge atom (except GeH_3_F···NCH and SiH_3_F···NCH). These results show that the assertion that tetrel bonds are just another category of non-covalent interactions is not correct, at least if this affirmation is solely based on the value of the bond order between the two fragments involved. On the other hand, it is apparent from Figure 2 that there is a gap in the center of the $V_{\mathrm{xc}}^{\mathrm{AB}}$ versus $\delta^{\mathrm{AB}}$ trend. It is possible that the reason for this gap is not the representativeness of the sample, although a wider exploration of complexes with the same electrophilic fragments as the ones used here but with many other electron-pair rich systems would be necessary to confirm this.

Since the exchange-correlation density can not be rigorously defined in DFT, the concept of delocalization index does not have a solid physical basis in that context. Nonetheless, the DFT $\delta^{AB}$ s can be formally calculated from the Kohn–Sham determinant of the system. Their values also appear in Table 2. In all cases, $\delta^{AB}\left(\mathrm{DFT}\right)>\delta^{AB}\left(\mathrm{CCSD}\right)$; i.e., DFT tends to exacerbate the bond order between the fragments A and B. Thus, the assertion of the above paragraph relative to the classification of tetrel bonds as covalent or non-covalent interactions becomes reinforced when DFT is used to obtain the delocalization indexes.

Adding the $V_{\mathrm{cl}}^{\mathrm{AB}}$ and $V_{\mathrm{xc}}^{\mathrm{AB}}$ energies, we obtain $E_{\mathrm{int}}^{\mathrm{AB}}$, the total interaction energy between A and B. Taking into account our previous comments regarding the relative (and comparable) values $V_{\mathrm{cl}}^{\mathrm{AB}}$ and $V_{\mathrm{xc}}^{\mathrm{AB}}$, it is clear that $E_{\mathrm{int}}^{\mathrm{AB}}$ is more stabilizing than each of its two contributions individually. Were it not for the damping and destabilizing effect caused by the deformation energies, some of the fragments of the investigated complexes would be strongly binded. However, since $E_{\mathrm{bind}}^{\mathrm{AB}}=E_{\mathrm{def}}^{A}+E_{\mathrm{def}}^{B}+E_{\mathrm{int}}^{\mathrm{AB}}$, the final values of $E_{\mathrm{bind}}^{\mathrm{AB}}$ (second or third column in Table 1) are, with some exceptions, relatively small.

The sum of the deformation energies of the fragments plus the exchange interaction energy ($E_{\mathrm{xr}}^{\mathrm{AB}}$, Equation (10)) plays, in the IQA method [57,69], a role very similar to the sum of the Pauli repulsion energy, $\Delta E_{\mathrm{Pauli}}$, plus the orbital relaxation term, $\Delta E_{\mathrm{orb}}$, in the energy decomposition analysis (EDA) method [66,67,68]. Actually, when the fragments interact but overlap very weakly, $E_{\mathrm{cl}}^{\mathrm{AB}}$ tends to the classical electrostatic EDA term, $V_{\mathrm{elstat}}^{\mathrm{AB}}$, and $E_{\mathrm{xr}}^{\mathrm{AB}}$ must converge to $\Delta E_{\mathrm{Pauli}}$. The values of $E_{\mathrm{xr}}^{\mathrm{AB}}$ in Table 1 are positive in all cases except in the CS_2_···CO complex, where it is marginally negative (−0.31 Kcal/mol, not very significant due to the inherent inaccuracy of the IQA numerical integration) and in the GeO_2_···Br^-^ system (−16.9 Kcal/mol). The negative and not negligible value of $E_{\mathrm{xr}}^{\mathrm{AB}}$ in this last case highlights that the hypothesis of weak overlap between both fragments, necessary for $E_{\mathrm{xr}}^{\mathrm{AB}}≃\Delta E_{\mathrm{Pauli}}$, is very far from being satisfied in GeO_2_···Br^-^. The high $\delta^{\mathrm{AB}}$ value in Table 2, fairly similar to that of a typical simple covalent bond (and the largest of all calculated delocalization indexes), further reinforces this claim. There is a very clear separation between the complexes containing Si or Ge in the acceptor fragment and those in which this fragment is CH_4_, CH_3_F, CO_2_, or CS_2_. When the element of group 14 is C, $E_{\mathrm{xr}}^{\mathrm{AB}}$ is never greater than 6.0 Kcal/mol, whereas $E_{\mathrm{xr}}^{\mathrm{AB}}$ in the complexes with SiO_2_ is several tens of Kcal/mol and as large as 127.83 Kcal/mol in the SiH_4_···F^-^ the complex. Although we have already commented that when $E_{\mathrm{xr}}^{\mathrm{AB}}$ is very large and positive, $E_{\mathrm{cl}}^{\mathrm{AB}}$ also happens to be large and negative, the compensation is not perfect, and consequently, the values of $E_{\mathrm{bind}}^{\mathrm{AB}}$ for the complexes containing Si or Ge are, in general, the greater. In the case of GeO_2_···Br^-^, both $E_{\mathrm{xr}}^{\mathrm{AB}}$ and $V_{\mathrm{cl}}^{\mathrm{AB}}$ are negative, and this makes the value of the binding energy for it almost the most stabilizing of all the systems analyzed, with the exception of SiO_2_···Br^-^. Negative exchange-repulsion terms can only be interpreted as being due to strong covalency.

Among the Si- and Ge-containing complexes, SiH_3_F···NCH and GeH_3_F···NCH present some peculiarities. Their inter-fragment Pauli exchange-repulsion energies are very small (3.74 and 4.66 Kcal/mol, respectively), just like their classical (−5.06 and −5.92 Kcal/mol), exchange-correlation (−16.68 and −16.04 Kcal/mol), and deformation energy (8.44, 10.29, and 9.20, 9.77 Kcal/mol) contributions. In fact, taking a look at the C-containing complexes in the acceptor fragment, we observe that when the electron donor group is NCH, all of these energy components tend to be lower than in the case of other acceptor groups. Two examples of this are the classical interaction energy, $V_{\mathrm{cl}}^{\mathrm{AB}}$, in the CH_3_F···NCH and CO_2_···NCH complexes, with −1.94 and −2.28 Kcal/mol, respectively. These numbers should not lead us to believe that the interactions between two individual atoms, one of each fragment, are also small. For instance, the C···N and C···C classical energies in CO_2_···NCH are −366.04 and 284.32 Kcal/mol, respectively, and the O···N and O···C energies are about 179.06 and −139.57 Kcal/mol, respectively. When the full C···NCH $V_{\mathrm{cl}}^{\mathrm{AB}}$ interaction is computed, its value becomes −39.00 Kcal/mol, an order of magnitude lower than the figures commented above. If all the interactions between the atoms of the electron donor fragment and those of the acceptor fragment are added together, the quantity −1.94 Kcal/mol that appears in Table 1 is obtained. This type of analysis can be done with the classical components of the interaction of any of the systems in the Table and the conclusions would be the same: individual atom-atom energies can be, in general, quite large. However, due to the almost electroneutrality of the fragments in many cases, they tend to cancel out in the final picture. As we have recently stressed, the meaning of Coulombic terms in the computation of intermolecular or interfragment energies is simple, but a considerable effort is still necessary before it is fully understood. As a final note, we want to emphasize that while measure of the intrinsic bond strength between two molecules, atoms, or fragments can be obtained from the plain $E_{\mathrm{int}}^{\mathrm{AB}}$ values [82], the calculation of the total binding energy unavoidably requires that the deformation energies be added to $E_{\mathrm{int}}^{\mathrm{AB}}$.

## 5. Conclusions

The interacting quantum atoms (IQA) methodology has been used to carry out a detailed energy partition of about thirty tetrel bonds formed between different electron-acceptor fragments (A) containing a C, Si, or Ge atom, and several neutral and anionic electron-donor fragments (B). The geometries of all the complexes were fully optimized at the DFT level, and all subsequent IQA analyses were performed at the CCSD level.

Almost every energetic quantity contributing to the total binding energy between A and B, $E_{\mathrm{bind}}^{\mathrm{AB}}$, is separated in the IQA method into intra-atomic and interatomic components. Adding together all the one- and two-center terms belonging to a given fragment, one obtains its net- or self-energy, $E_{\mathrm{net}}^{R}$ (R=A,B). When the total energies of the isolated fragments are computed at the same computational level as the complexes and subtracted from $E_{\mathrm{net}}^{R}$, the fragment deformation energies $E_{\mathrm{def}}^{R}$ appear. Their computed values are systematically positive, and the greater or smaller value of each $E_{\mathrm{def}}^{R}$ gives a measure of the degree of electronic reorganization suffered by the fragment upon complex formation. Due to their positive values, the deformation energies are destabilizing contributions to $E_{\mathrm{bind}}^{\mathrm{AB}}$. Complexes containing a C atom in the acceptor fragment are those with the smaller deformation energies, and those that contain a Si atom have greater $E_{\mathrm{def}}^{R}$ values than their analogues with germanium.

A detailed analysis of all IQA energy contributions leads us to conclude that, overall, the IQA energy quantities obtained for the complexes in which the charge-acceptor fragment (A) contains a C atom are smaller than when the atom of group 14 is Si, which, in general but with some exceptions, are usually greater than when the complex contains Ge instead of Si. In agreement with several authors, there are plenty of examples of tetrel interactions that can hardly be classified as non-covalent interactions. In some extreme cases, like in the GeO_2_···Br^-^ system, all real space indicators point toward a standard strong polar-covalent interaction. This situation is similar to that found in other recently defined bonds, where a full window of interaction energies going from very weak to considerably strong links have been found.

The IQA energy partition method used in this work is fully framed in the context of quantum chemical topology. Among its possible advantages over other existing schemes, its orbital invariance is possibly the most important of all. The IQA method can be applied independently of the electronic structure method used to construct the wave function that describes the molecular system under study. Accurate electronic structure methods, such as full interaction configuration (full-CI), multireference singles and doubles interaction configuration (MR-CISD), or the CCSD method used in this work, can be applied as easily as a mean field scheme, such as the Hartree–Fock method. Actually, in order for IQA to be used, it only requires the knowledge of the one-particle and (diagonal) two-particle density matrices, although molecular descriptions at the DFT level are also possible in the IQA context. Finally, although IQA has, to date, been applied almost exclusively in the ground electronic state, we have also recently started to use it in excited electronic states [83,84]. 

## Figures and Tables

**Table 1:** Columns 2–7 collect the binding energy obtained from Equations (5), $E_{\mathrm{bind}}\left(\mathrm{CCSD}\right)\equiv \left(\mathrm{CCSD}\right)$, and (8), $E_{\mathrm{bind}}\left(\mathrm{IQA}\right)\equiv \left(\mathrm{IQA}\right)$, its difference ($\mathrm{diff}=E_{\mathrm{bind}}\left(\mathrm{IQA}\right)-E_{\mathrm{bind}}\left(\mathrm{CCSD}\right)$), and the total interacting quantum atom (IQA) integrated charges, $Q_{A}$, $Q_{B}$, and $Q=Q_{A}+Q_{B}$ Columns 8–14 show the different contributions to $E_{\mathrm{bind}}\left(\mathrm{IQA}\right)$, according to Equations (8)–(11). Energy units in (Kcal/mol); CCSD—coupled cluster singles and doubles).

System (A···B)	(CCSD)	(IQA)	diff	$Q_{A}$	$Q_{B}$	*Q*	$E_{\mathrm{def}}^{A}$	$E_{\mathrm{def}}^{B}$	$V_{\mathrm{cl}}^{\mathrm{AB}}$	$V_{x}^{\mathrm{AB}}$	$V_{\mathrm{xc}}^{\mathrm{AB}}$	$E_{\mathrm{int}}^{\mathrm{AB}}$	$E_{\mathrm{xr}}^{\mathrm{AB}}$
CH_4_···F^-^	−3.42	−3.41	0.01	−0.0112	−0.9891	−1.0003	8.42	3.77	−6.27	−8.46	−9.33	−15.60	3.73
SiH_4_···F^-^	−60.17	−59.87	0.31	−0.0426	−0.9579	−1.0005	123.02	87.24	−183.63	−82.43	−86.50	−270.13	127.83
GeH_4_···F^-^	−40.17	−38.89	1.28	−0.0830	−0.9170	−1.0001	79.50	46.57	−92.28	−69.62	−72.69	−164.97	56.46
CH_3_F···NCH	−1.52	−1.36	0.16	−0.0033	0.0032	−0.0000	3.37	3.47	−1.94	−5.53	−6.25	−8.19	1.31
CH_3_F···N_3_−	−9.21	−8.78	0.44	−0.0200	−0.9789	−0.9989	11.18	5.05	−8.47	−14.46	−16.54	−25.01	1.77
CH_3_F···NCO^-^	−9.89	−9.69	0.21	−0.0188	−0.9809	−0.9997	10.11	6.32	−11.73	−12.96	−14.39	−26.12	3.47
CH_3_F···OCN^-^	−8.37	−8.31	0.06	−0.0147	−0.9854	−1.0001	10.24	5.51	−9.28	−12.97	−14.78	−24.06	2.78
SiH_3_F···NCH	−3.06	−3.02	0.04	−0.0052	0.0043	−0.0008	8.44	10.29	−5.06	−14.99	−16.68	−21.75	3.74
SiH_3_F···N_3_−	−40.91	−41.45	−0.54	−0.0678	−0.9282	−0.9960	73.85	70.33	−103.27	−77.13	−82.36	−185.64	67.05
GeH_3_F···NCH	−3.65	−2.99	0.66	−0.0031	0.0041	0.0010	9.20	9.77	−5.92	−14.31	−16.04	−21.96	4.66
GeH_3_F···N_3_−	−36.72	−36.31	0.40	−0.1110	−0.8878	−0.9988	46.32	39.64	−53.01	−65.10	−69.26	−122.27	20.86
CO_2_···NCH	−1.60	−2.08	−0.48	−0.0056	0.0059	0.0003	3.55	4.13	−2.28	−6.58	−7.47	−9.76	1.09
CO_2_···N_3_−	−6.79	−6.46	0.34	−0.0236	−0.9757	−0.9994	12.43	8.07	−9.87	−15.31	−17.09	−26.96	5.20
CO_2_···CN^-^	−8.25	−8.64	−0.39	−0.0564	−0.9428	−0.9992	14.77	11.78	−10.12	−23.26	−25.07	−35.19	3.29
CO_2_···CO	−0.65	−1.14	−0.49	−0.0058	0.0058	0.0000	2.71	2.88	−0.76	−5.31	−5.97	−6.73	0.28
CO_2_···CS	−1.29	−1.83	−0.54	−0.0105	0.0096	−0.0008	3.84	4.19	−1.68	−7.27	−8.19	−9.87	0.77
CO_2_···Br^-^	−5.60	−4.99	0.62	−0.0401	−0.9600	−1.0001	9.08	6.79	−4.97	−14.01	−15.89	−20.86	1.87
CO_2_···Kr	−0.48	+0.03	0.52	−0.0035	0.0028	−0.0007	0.58	2.33	−0.08	−2.29	−2.79	−2.88	0.61
CO_2_···NC^-^	−8.34	−8.89	−0.55	−0.0333	−0.9662	−0.9996	14.29	10.83	−12.70	−19.43	−21.31	−34.01	5.69
CO_2_···NH_3_	−2.09	−2.41	−0.31	−0.0158	0.0155	−0.0003	6.63	7.39	−4.12	−11.06	−12.32	−16.43	2.97
CO_2_···OC	−0.44	−0.91	−0.47	0.0001	−0.0000	0.0001	1.11	1.40	−0.57	−2.39	−2.85	−3.42	0.12
CO_2_···OH_2_	−2.24	−2.52	−0.28	−0.0051	0.0052	0.0000	5.42	5.87	−3.86	−8.86	−9.94	−13.81	2.43
CO_2_···SH^-^	−3.99	−4.32	−0.33	−0.0244	−0.9758	−1.0003	6.64	3.96	−3.67	−9.68	−11.25	−14.93	0.93
SiO_2_···NCH	−22.43	−21.00	1.42	−0.0174	0.0185	0.0010	57.82	77.23	−89.17	−62.94	−66.89	−156.06	72.12
SiO_2_···CO	−9.61	−8.69	0.92	−0.0112	0.0114	0.0002	49.33	69.34	−57.46	−66.28	−69.91	−127.37	52.39
SiO_2_···CS	−30.17	−28.78	1.39	−0.0082	0.0086	0.0003	75.50	92.86	−107.86	−85.39	−89.29	−197.15	82.98
SiO_2_···Br^-^	−78.06	−76.19	1.87	−0.1552	−0.8433	−0.9985	60.08	84.17	−134.94	−79.98	−85.51	−220.45	64.27
CS_2_···CO	−0.77	−0.86	−0.09	−0.0008	0.0010	0.0002	0.96	1.64	−0.09	−2.90	−3.36	−3.45	−0.31
CS_2_···CS	−0.95	−0.46	0.49	−0.0007	0.0006	−0.0001	3.95	3.57	−0.40	−6.37	−7.57	−7.98	1.14
CS_2_···OH_2_	−1.48	−1.97	−0.40	0.0012	−0.0003	0.0009	2.44	3.17	−1.88	−4.99	−5.70	−7.58	0.61
GeO_2_···Br^-^	−65.37	−65.29	0.08	−0.2923	−0.7068	−0.9991	23.17	48.63	−44.92	−88.70	−92.17	−137.09	−16.90

**Table 2:** CCSD and density functional theory (DFT) delocalization indexes, $\delta^{\mathrm{AB}}$.

A···B	CCSD	DFT	A···B	CCSD	DFT
CH_4_···F^-^	0.0910	0.1281	CO_2_···Br^-^	0.1649	0.2030
SiH_4_···F^-^	0.6142	0.7611	CO_2_···Kr	0.0299	0.0357
GeH_4_···F^-^	0.5431	0.6878	CO_2_···NC^-^	0.1871	0.2364
CH_3_F···NCH	0.0602	0.0752	CO_2_···NH_3_	0.1137	0.1405
CH_3_F···N_3_−	0.1564	0.1877	CO_2_···OC	0.0274	0.0338
CH_3_F···NCO^-^	0.1328	0.1672	CO_2_···OH_2_	0.0878	0.1086
CH_3_F···OCN^-^	0.1378	0.1700	CO_2_···SH^-^	0.1178	0.1425
SiH_3_F···NCH	0.1526	0.1837	SiO_2_···NCH	0.5048	0.6375
SiH_3_F···N_3_−	0.6431	0.7535	SiO_2_···CO	0.5439	0.7054
GeH_3_F···NCH	0.1479	0.1770	SiO_2_···CS	0.6823	0.9346
GeH_3_F···N_3_−	0.5702	0.6671	SiO_2_···Br^-^	0.6868	0.8568
CO_2_···NCH	0.0694	0.0860	CS_2_···CO	0.0368	0.0431
CO_2_···N_3_−	0.1546	0.1919	CS_2_···CS	0.0803	0.0930
CO_2_···CN^-^	0.2320	0.2910	CS_2_···OH_2_	0.0560	0.0677
CO_2_···CO	0.0596	0.0726	GeO_2_···Br^-^	0.7631	0.9514
CO_2_···CS	0.0806	0.0980			

## Abbreviations

The following abbreviations are used in this manuscript:

QTAIM	Quantum theory of atoms in molecules
IQA	Interacting quantum atoms
NBO	Natural bond orbital
MP2	Second-order Møller–Plesset
MEP	Molecular electrostatic potential
EDA	Energy decomposition analysis
DFT	Density functional theory
CCSD	Singles and doubles coupled cluster
HF	Hartree–Fock
FNO	Frozen natural orbital
BSSE	Basis set superposition error
EOM	Equation of motion
full-CI	Full interaction configuration
MR-CISD	Multireference singles and doubles interaction configuration
